# Identification of Bacteria in Blood Culture Broths Using Matrix-Assisted Laser Desorption-Ionization Sepsityper™ and Time of Flight Mass Spectrometry

**DOI:** 10.1371/journal.pone.0023285

**Published:** 2011-08-16

**Authors:** Jen Kok, Lee C. Thomas, Thomas Olma, Sharon C. A. Chen, Jonathan R. Iredell

**Affiliations:** 1 Centre for Infectious Diseases and Microbiology Laboratory Services, Institute of Clinical Pathology and Medical Research, Westmead Hospital, Westmead, New South Wales, Australia; 2 Centre for Research Excellence in Critical and Emerging Infectious Diseases, University of Sydney, Westmead Hospital, Westmead, New South Wales, Australia; Charité, Campus Benjamin Franklin, Germany

## Abstract

Matrix-assisted laser desorption ionization time of flight mass spectrometry (MALDI-TOF MS) is a novel method for the direct identification of bacteria from blood culture broths. We evaluate for the first time, the performance of the MALDI Sepsityper™ Kit and MS for the identification of bacteria compared to standard phenotypic methods using the manufacturer's specified bacterial identification criteria (spectral scores ≥1.700–1.999 and ≥2.000 indicated identification to genus and species level, respectively). Five hundred and seven positive blood culture broths were prospectively examined, of which 379 (74.8%; 358 monomicrobial, 21 polymicrobial) were identified by MALDI-TOF MS; 195 (100%) and 132 (67.7%) of 195 gram-positive; and 163 (100%) and 149 (91.4%) of 163 gram-negative organisms from monomicrobial blood cultures were correctly identified to genus and species level, respectively. Spectral scores <1.700 (no identification) were obtained in 128/507 (25.2%) positive blood culture broths, including 31.6% and 32.3% of gram-positive and polymicrobial blood cultures, respectively. Significantly more gram-negative organisms were identified compared to gram-positive organisms at species level (p<0.0001). Five blood cultures were misidentified, but at species level only; including four monomicrobial blood cultures with *Streptococcus oralis*/*mitis* that were misidentified as *Streptococcus pneumoniae*. Positive predictive values for the direct identification of both gram-positive and gram-negative bacteria from monomicrobial blood culture broths to genus level were 100%. A diagnostic algorithm for positive blood culture broths that incorporates gram staining and MALDI-TOF MS should identify the majority of pathogens, particularly to genus level.

## Introduction

Bloodstream infections (BSIs) are a significant cause of morbidity and mortality in hospitals. In the United States, septicemia has consistently featured in the top 10 causes of death, accounting for 35,587 deaths (11.6 per 10^5^ population) in 2009 alone [1]. In Australia, in-hospital mortality for patients presenting with septic shock ranges from 23.1–27.6% [2], [3]. In the first six hours of septic shock, each hour of delay in initiating effective antimicrobial therapy following the onset of hypotension is associated with a reduction in average survival by 7.6% [4]. Rapid, accurate identification of the etiologic pathogen is critical for guiding effective antimicrobial therapy and improving patient outcomes, and for reducing length of hospitalization and hospital costs [5].

Traditional phenotypic based diagnostic methods for BSIs require the detection of bacterial growth in blood culture broths, followed by species identification and antimicrobial susceptibility testing (turnaround time 24–48 hours after initial growth). Pathogens with fastidious growth requirements and those difficult to identify by phenotypic methods require more time for identification. Rapid nucleic acid amplification methods such as real-time PCR using melting curve analysis, multiplex PCR, fluorescence in situ hybridization (FISH) and peptide nucleic acid-FISH (PNA-FISH) have been used to detect pathogens in blood cultures including *Staphylococcus aureus*, *Enterococcus faecalis* and *Candida albicans* [6], [7], [8]. These assays, however, only target specific organisms; require technical expertise; and specimens are usually processed in batches. Turnaround times are up to 6 hours.

The matrix assisted laser desorption-ionization time of flight mass spectrometry (MALDI-TOF MS) is a novel method for the direct identification of pathogens in blood culture broths, with results available within 2 hours. Although it does not provide antimicrobial susceptibility data (with the exception of methicillin resistant *Staphylococcus aureus* [MRSA]), it has good potential to guide empirical antimicrobial choice in the treatment of BSIs, yet there remain technical variables that may affect test performance.

Different methods for the preparation of blood culture broths prior to MS analysis have been utilized. Some investigators have employed an intact cell method, while others have extracted bacterial proteins using different solutions, including home-made ammonium chloride, trifluoroacetic acid (TFA), formic acid and acetonitrile; one study noted improved bacterial identification when formic acid instead of TFA was used [9], [10], [11]. There is also significant variation in the number of washing/centrifugation steps [9]. Different interpretive criteria have also been applied for bacterial identification, with some investigators accepting identification when consecutive spectral scores were greater than a pre-defined threshold on repeated analysis, despite the threshold being lower than the manufacturer's recommendations [11]. The lack of protocol standardization may contribute to reported differences in the performance of MALDI-TOF MS in the direct identification of pathogens from blood culture broths.

More recently, the MALDI Sepsityper™ Kit (Bruker Daltonics Inc., Billerica, MA) has been introduced to standardize the processing of blood culture broths prior to MS identification. This system outlines a specific protocol and provides all the reagents needed. Herein, we evaluate the performance of the MALDI Sepsityper™ Kit and MS for the direct identification of blood culture broths known to contain bacteria.

## Methods

### Blood cultures

From March to April 2011, blood culture broths (BACTEC™ Plus Aerobic/F and Lytic/10 Anaerobic/F blood culture media; Becton Dickinson, Franklin Lakes, NJ) collected from patients with suspected sepsis identified as positive by the BACTEC™ FX blood culture system were prospectively analyzed using MALDI-TOF MS (Bruker Daltonics Inc.) and standard phenotypic identification methods in parallel.

### Phenotypic identification methods

Gram stains were performed on positive blood culture broths, prior to subculture on horse blood, chocolate, blood/haemin/vitamin K and MacConkey agars (Oxoid, Thermo Fisher Australia Pty Ltd, Thebarton, Adelaide, South Australia) as appropriate, and incubated at 37°C in CO_2_ for 18–24 hours, or anaerobically for 48 hours. Isolates were then identified using the Phoenix automated microbiology system (Phoenix; BD Diagnostic Systems, Sparks, MD). Gram-positive cocci resembling staphylococci on gram stain were subjected to the tube coagulase (Rabbit Plasma, Bio-Rad, Marnes-la-Coquette, France) test, and staphylococcal isolates were tested with i) staphylococcal latex agglutination (BactiStaph, Remel Inc., Lenexa, KS) test, ii) DNAse test and iii) in some cases, the Phoenix system for confirmation of identification. Where identification by the Phoenix system was inconclusive or suspected to be incorrect, phenotypic identification of isolates was performed using API (bioMérieux, Marcy-l'Etoile, France) or RapID™ ANA II System (Remel Inc., Lenexa, KS).

### MALDI Sepsityper™ kit

Sepsityper™ Kit preparation of positive blood cultures broths was performed according to the manufacturer's instructions. Briefly, 200 µL of lysis buffer was added to 1 mL of positive blood culture fluid in a reaction tube. The tube was vortexed for 10 seconds prior to centrifugation at 13,000 rpm for 1 minute. The supernatant was then discarded, the pellet suspended with 1 mL of washing buffer, and re-centrifuged at 13,000 rpm for a further minute. The supernatant was discarded once more, and the pellet resuspended in 300 µL of deionized water, and 900 µL of ethanol was added.

### Ethanol/formic acid extraction

The suspension obtained following sample preparation as described above was centrifuged at 13,000 rpm for 2 minutes, and the supernatant discarded. The pellet was centrifuged for another 2 minutes prior to removal of residual ethanol. Sequentially, 2–50 µL each of formic acid (70% v/v) and 100% acetonitrile was added to the pellet (depending on pellet size), and thoroughly mixed after each reagent was added. The resuspension was centrifuged again at 13,000 rpm for another 2 minutes, and 1 µL of the supernatant was spotted onto the steel target plate. Analysis was performed following air-drying of 1 µL HCCA (α-cyano-4-hydroxycinnamic acid) matrix solution placed onto the dried sample spot in duplicate.

### Mass spectrometry fingerprinting

Mass spectra were generated with the Microflex LT mass spectrometer operated by the MALDI-Biotyper automation control (Bruker Daltonics Inc.). Three hundred shots per sample spot were acquired using the recommended instrument settings for bacterial identification (linear positive mode, 60 Hz laser frequency, 20 kV acceleration voltage, 16.7 kV IS2 voltage, 170 ns extraction delay, and 2,000 to 20,137 m/z range). Manual and automated spectrum processing (smoothing, baseline subtraction and peak picking) and species identification were done with the MALDI-Biotyper 2.0 application. The software compares acquired sample spectra to reference spectra in the provided database and compiles a list of best matching database records. The degree of spectral concordance is expressed as a logarithmic identification score and interpreted according to the manufacturer's instructions: scores ≥2.300 indicated species identification with a high level of confidence, ≥2.000 indicated species identification, 1.700–1.999 indicated genus identification, and <1.700, no identification.

## Results

From March to April 2011, a total of 507 positive blood cultures broths were identified by the Bactec FX automated blood culture system, of which 476 (93.9%) were monomicrobial and 31 (6.1%) were polymicrobial. This study focused on bacteria only; yeasts were excluded from the analysis. Identification to genus (spectral scores ≥1.700) and species (spectral scores ≥2.000) level was obtained in 379/507 (74.8%) and 301/507 (59.4%) blood culture broths respectively. Five of 379 (1.3%) positive blood culture broths were misidentified.

### Monomicrobial blood cultures

Using standard phenotypic methods, gram-positive, gram-negative and anaerobic bacteria were identified in 285 (59.9%), 187 (39.3%) and 4 (0.8%) of 476 broths respectively. Tables 1 and 2 show the results of identification of gram-positive, and gram-negative monomicrobial blood culture broths respectively.

**Table 1:** MALDI-TOF MS identification of monomicrobial Gram-positive bacteremia compared to standard phenotypic identification.

Organism	Samples (n)	Not identified (score <1.700) (n,%)	Misiden-tified (n,%)	Identified to genus level (score 1.700–1.999) (n,%)	Identified to species level (score ≥2.000) (n,%)	High level identification (score ≥2.300) (n,%)
Staphylococcal spp. a	204	59 (28.9%)	0	145 (71.1%)	99 (48.5%)	20 (9.8%)
*S. aureus* (total)	54	5 (9.3%)	0	49 (90.7%)	47 (87%)	14 (25.9%)
MSSA	47	4 (8.5%)	0	43 (91.5%)	41 (87.2%)	10 (21.3%)
MRSA	7	1 (14.3%)	0	6 (85.7%)	6 (85.7%)	4 (57.1%)
CNS	150	54 (36%)	0	96 (64%)	52 (34.7%)	6 (4%)
Streptococcal spp. a	33	9 (29%)	4 (12.9%)^c^	24 (72.7%)	15 (48.4%)	7 (22.6%)
*S. pyogenes*	4	0	0	4 (100%)	4 (100%)	2 (50%)
*S. pneumoniae*	2	1 (50%)	0	1 (50%)	1 (50%)	0
*S. dysgalactiae*	5	1 (20%)	0	4 (80%)	1 (20%)	0
*S. oralis/mitis*	9	4 (44.4%)	4 (44.4%)^c^	5 (55.6%)	0	0
*S. mutans*	1	0	0	1 (100%)	0	0
*S. salivarius*	3	2 (66.7%)	0	1 (33.3%)	1 (33.3%)	0
*S. anginosus*	1	1 (100%)	0	0	0	0
*S. agalactiae*	6	0	0	6 (100%)	6 (100%)	5 (83.3%)
*S. gordonii*	2	0	0	2 (100%)	2 (100%)	0
Enteroococcal spp. a	13	7 (53.8%)	0	6 (46.2%)	5 (38.5%)	1 (7.7%)
*E. faecalis*	9	4 (44.4%)	0	5 (55.6%)	4 (44.4%)	1 (11.1%)
*E. faecium*	2	1 (50%)	0	1 (50%)	1 (50%)	0
*Enterococcus* spp.^a^ (non-faecalis/faecium)	2	2 (100%)	0	0	0	0
*Micrococcus* spp.^a^	6	1 (16.7%)	0	5 (83.3%)	3 (50%)	0
*Gemella morbillorum*	1	0	0	1 (100%)	0	0
*Lactococcus* spp.^a^	4	0	0	4 (100%)	3 (75%)	0
*Rhodococcus equi*	1	1 (100%)	0	0	0	0
*Rothia mucilaginosa*	3	0	0	3 (100%)	2 (66.7%)	0
Diphtheroids	8	6 (75%)	0	2 (25%)	2 (25%)	0
*Bacillus* spp.^a^	3	1 (33.3%)	0	2 (66.7%)	1 (33.3%)	0
*Propionibacterium* spp.^a^	6	4 (66.7%)	0	2 (33.3%)	1 (16.7%)	0
*Lactobacillus catenaeformis*	1	0	0	1 (100%)	1 (100%)	0
*Microbacterium* spp.^a^	1	1 (100%)	0	0	0	0
*Leuconostoc* spp.^a^	1	1 (100%)	0	0	0	0
**Gram-positive total**	**285**	**90 (31.6%)**	**4 (1.4%)**	**195 (68.4%)**	**132 (46.3%)**	**28 (9.8%)**

a species.

b includes *S. epidermidis*, *S. warneri*, *S. haemolyticus*, *S. lugdunensis*, *S. capitis* and *S. hominis*.

c misidentification to species level only.

**Table 2:** MALDI-TOF MS identification of monomicrobial Gram-negative bacteremia compared to standard phenotypic identification.

Organism	Samples (n)	Not identified (score <1.700) (n,%)	Identified to genus level (score 1.700–1.999) (n,%)	Identified to species level (score ≥2.000) (n,%)	High level identification (score ≥2.300) (n,%)
*Escherichia coli*	102	5 (4.9%)	97 (95.1%)	96 (94.1%)	75 (73.5%)
*Klebsiella pneumoniae*	11	1 (9.1%)	10 (90.9%)	10 (90.9%)	4 (36.4%)
*Klebsiella oxytoca*	10	2 (20%)	8 (80%)	8 (80%)	4 (40%)
*Klebsiella kristinae*	1	1 (100%)	0	0	0
*Salmonella typhi*	6	0	6 (100%)	0	0
*Salmonella paratyphi*	5	0	5 (100%)	0	0
*Enterobacter cloacae*	7	0	7 (100%)	6 (85.7%)	1 (14.3%)
*Citrobacter freundii*	2	0	2 (100%)	2 (100%)	0
*Citrobacter koseri*	2	1 (50%)	1 (50%)	1 (50%)	0
*Citrobacter amalonaticus*	2	0	2 (100%)	2 (100%)	0
*Morganella morganii*	4	0	4 (100%)	4 (100%)	2 (50%)
*Proteus mirabilis*	2	0	2 (100%)	2 (100%)	0
*Serratia marcescens*	3	0	3 (100%)	3 (100%)	0
*Aeromonas hydrophila*	1	0	1 (100%)	1 (100%)	0
*Aeromonas veronii*	1	1 (100%)	0	0	0
*Pseudomonas aeruginosa*	12	1 (8.3%)	11 (91.7%)	11 (91.7%)	2 (16.7%)
*Pseudomonas stutzeri*	1	1 (100%)	0	0	0
*Pseudomonas putida*	1	0	1 (100%)	0	0
*Acinetobacter baumannii*	2	1 (50%)	1 (50%)	1 (50%)	0
*Acinetobacter lwoffii*	1	1 (100%)	0	0	0
*Stenotrophomonas maltophilia*	5	4 (80%)	1 (20%)	1 (20%)	0
*Sphingomonas paucimobilis*	1	1 (100%)	0	0	0
*Achromobacter xylosoxidans*	3	2 (66.7%)	1 (33.3%)	1 (33.3%)	0
*Brevundimonas vesicularis*	1	1 (100%)	0	0	0
*Roseomonas spp.* a	1	1 (100%)	0	0	0
**Gram-negative total**	**187**	**24 (12.8%)**	**163 (87.2%)**	**149 (79.7%)**	**88 (47.1%)**

a species.

Of 195 gram-positive organisms identified by MALDI-TOF MS, 195 (100%) and 132 (67.7%) blood culture broths were identified to genus and species level respectively. Significantly more broths with gram-negative organisms were correctly identified compared to gram-positive organisms at species (91.4% [149/163] vs. 67.7%, p<0.0001) level. High-level identification (spectral scores ≥2.300) was obtained for 88/187 (47.1%) broths with gram-negative isolates, but only 28/285 (9.8%) broths with gram-positive isolates. MALDI-TOF MS was unable to identify 9.3% and 36% of broths positive for *S. aureus* and coagulase negative staphylococci respectively. However, all *S. aureus* or coagulase negative staphylococci that were identified by MALDI-TOF MS had concordant identification by phenotypic methods.

All four broths containing anaerobic organisms (one each of *Bacteroides fragilis*, *Prevotella melaninogenica*, *Peptostreptococcus* species and *Finegoldia magna*) were not identified by MALDI-TOF MS. 53.8% of enterococci and 65% of gram-positive rods were also not identified. Four (0.8%) bacteremias caused by monomicrobial isolates were misidentified, but at species level only (four *Streptococcus oralis*/*mitis* were misidentified as *Streptococcus pneumoniae*). A single discrepancy was noted between MALDI-TOF MS and phenotypic identification of a gram-negative bacterium, with MALDI-TOF MS identifying an isolate that was established to be *Pseudomonas putida* by Phoenix as *Pseudomonas fulva*. Positive predictive values for the direct identification of both gram-positive and gram-negative bacteria from monomicrobial blood culture broths to genus level were 100%.

### Polymicrobial blood cultures

Of 31 polymicrobial blood cultures broths, 10 (32.3%) were not identified and 1 (3.2%) was misidentified (at species level). In the remaining 20 polymicrobial blood cultures, MALDI-TOF MS was able to identify one pathogen, despite the gram stain suggesting the presence of more than one organism in some instances. One blood culture with *Citrobacter freundii* was identified by MALDI-TOF MS to genus level only. Two sets of blood cultures were independently collected from a single patient, from which *Klebsiella pneumoniae* and *Aeromonas hydrophila* were isolated. MALDI-TOF MS identified *A. hydrophila* in one set, but *A. veronii* in the other set of blood cultures. This was the only discrepant result between MALDI-TOF MS and Phoenix identification in polymicrobial bacteremias. The results of identification of polymicrobial bacteremia isolates are outlined in Table 3.

**Table 3:** MALDI-TOF MS identification of polymicrobial bacteremia compared to standard phenotypic identification.

Organisms	Samples (n)	Not identified (score <1.7000) (n,%)	Misidentified (n,%)	MALDI-TOF MS identification^a^
MRSA/*S. epidermidis*	1	0	0	*S. aureus*
MSSA/*P. aeruginosa*/*S. marcescens*	3	0	0	*S. aureus* (1), *S. marcescens* (2)
CNS^b^×2 types	6	1 (100%)	0	*S. epidermidis* (1), *S. hominis* (2), *S. capitis* (2)
CNS^b^/*S. epidermidis*	1	0	0	*S. epidermidis*
*S. epidermidis*/CNS^b^×2 types	1	0	0	*S. epidermidis*
CNS^b^/*Bacillus* spp/non-haemolytic *Streptococcus*	1	1 (100%)	0	-
CNS^b^/Diptheroid	1	1 (100%)	0	-
Group G *Streptococcus*/*A. baumannii*	1	1 (100%)	0	-
*S. oralis*/*mitis* group/*G. haemolysans*/*Veillonella* spp/*A. odontolyticus*	1	0	0	*G. haemolysans*
α-haemolytic/non-haemolytic *Streptococcus*	1	1 (100%)	0	-
α-haemolytic *Streptococcus*/CNS^b^	1	1 (100%)	0	-
*E. faecalis*/*P. aeruginosa*	1	0	0	*E. faecalis*
Diptheroid×2 types	1	1 (100%)	0	-
*E. coli*/*E. cloacae*	1	0	0	*E. coli*
*K. oxytoca*/*E. coli*	2	0	0	*E. coli* (2)
*K. pneumoniae/A. hydrophila*	2	0	1 (50%)^c^	*A. hydrophila* (1), *A. veronii* (1)
*E. cloacae*/*E. aerogenes*	1	0	0	*E. aerogenes*
*C. freundii*/*P. aeruginosa*	1	0	0	*Citrobacter* spp^d^
*P. aeruginosa*/*K. pneumoniae*	1	1 (100%)	0	-
*P. aeruginosa/S. maltophilia*	1	1 (100%)	0	-
*P. asaccharolyticus*/*P. granulosum*	1	1 (100%)	0	-
*A. lwoffii*/CNS^b^	1	0	0	*A. lwoffii*
**Total**	**31**	**10 (32.3%)**	**1 (3.2%)**	***-***

a number in parenthesis indicates number of blood cultures positive with organism identified.

b coagulase negative *Staphylococcus*.

c misidentification to species level only.

d species.

Table 4 outlines the performance characteristics of MALDI-TOF MS for the identification of commonly encountered blood culture pathogens in the present study compared to other contemporary studies where spectral scores were categorized in a similar manner [9], [10], [12].

**Table 4:** Comparison of MALDI-TOF MS identification of commonly encountered monomicrobial bloodstream infections in the present study against other contemporary studies.

Organism	Present study		Other contemporary studies a	
	Identification to genus level	Identification to species level	Identification to genus level	Identification to species level
**Gram positive organisms**				
*S. aureus*	90.7%	87%	30.6%–100%	5.5%–97.7%
CNS^b^	64%	34.7%	71.9%–84%	24%–96.7%
*Streptococcus* spp.^c^	72.7%	48.4%	50%–95.4%	31.8%–50%
*Enterococcus* spp.^c^	46.2%	38.5%	75%–76.5%	50%–70.6%
**Gram negative organisms**				
*E. coli*	95.1%	94.1%	97.6%–100%	93.3%–97.6%
*Klebsiella* spp.^c^	81.8%	81.8%	84.6%–100%	69.2%–100%
*P.aeruginosa*	91.7%	91.7%	85%–100%	85%–100%
Enterobacteriaceae	93.6%	85.4%	94.5%–97.9%	86.8%–94.5%
Non-fermenters	53.6%	50%	85.7%–100%	66.7%–85.7%

a composite data from [9], [10], [12].

b coagulase negative *Staphylococcus* (includes *S. epidermidis*).

c species.

### Ethics Statement

This study is not a research question, but a laboratory validation of a novel method for the specific identification of bacterial pathogens grown in artificial media. The study does not involve the collection or reporting of patient data, and no patient intervention occurred with the obtained results.

## Discussion

Identifying the etiologic pathogen, followed by antimicrobial susceptibility testing, is critical in the management of BSIs, as delays in effective antimicrobial therapy can adversely affect patient outcomes [4]. MALDI-TOF MS has significant potential over phenotypic methods, as it is able to detect bacterial pathogens directly from blood culture broths reliably and quickly. However, the performance of MALDI-TOF MS is affected by blood culture bottle type, methodology in sample preparation prior to MS analysis, and the interpretive criteria employed [9]–[15]. We report the performance of the MALDI Sepsityper™ Kit that provides all the reagents required to process blood culture broths according to a standardized protocol prior to MALDI-TOF MS analysis.

Using the manufacturer's specified criteria for bacterial identification, MALDI-TOF MS identified 74.8% of blood culture broths directly (spectral scores ≥1.700), of which only five (1%) were misidentified at species level. Reported concordance rates between MALDI-TOF MS and conventional phenotypic methods for the direct identification of bacteria in non-charcoal containing blood cultures vials vary between 64.8–97% at genus level and 31.8–91.1% at species level [9], [11], [12], [14], [16]. Our data is similar to contemporary studies, with 74.8% and 59.2% of organisms correctly identified to genus and species level respectively. Similar to previous investigators, we also found that MALDI-TOF MS was superior at identifying gram-negative compared to gram-positive organisms [9]–[11]. Some investigators have reported similar rates of non-identification (spectral scores <1.700) compared to ours (25.2%), while others have noted lower rates [10], [16], [17].

The discrepant *P. putida* identified by Phoenix that was identified by MALDI-TOF MS as *P. fulva* was likely to be the latter, although we did not perform 16S rRNA sequencing to confirm this. *P. fulva* and *P. putida* share similar phenotypic characteristics, and both have been placed within the *P. putida* complex following intrageneric structure reconstruction from nucleotide sequences of *gyrB* and *rpoD* genes [18]. Furthermore, several investigators have reported the misidentification of *P. fulva* as *P. putida* by automated identification systems [19], [20]. It is also not surprising that MALDI-TOF MS misidentified *A. hydrophila* as *A. veronii* in the polymicrobial bacteremia given the complex and controversial taxonomy of this genus. 16S rRNA gene sequencing was not attempted, as one result would have been discrepant, given that MALDI-TOF MS identified the pathogen as *A. hydrophila* in one set of blood cultures and *A. veronii* in the other. Furthermore, 16S rRNA sequencing alone may not resolve the discrepancy, as *gyrB* and *rpoD* gene sequencing has superseded 16S rRNA for improved identification of closely related *Aeromonas* species [21].

What is the current place of MALDI-TOF MS in the direct identification of blood culture pathogens? The rapid turnaround time of 2 hours for direct identification compares favourably with the timeframe (24–48 hours) required for identification by conventional methods after the initial “signalling” of organisms in blood cultures. This supports its use in the diagnostic algorithm of BSIs [22], [23], particularly for gram-negative bacteremias, where knowledge of the bacterial pathogen (and local resistance data) can have significant impact on the choice of antibiotics to be prescribed. In the case of gram-positive bacteremias, results of current phenotypic tests, including the morphological appearance of gram-positive cocci or bacilli on gram stain and the tube coagulase test (for gram-positive cocci suggestive of staphylococci), may be available within two hours, similar to the time required for MALDI-TOF MS analysis. In gram-positive organisms, both the choice of antibiotics for treatment, and antimicrobial resistance mechanisms, are more limited. As MALDI-TOF MS directly identifies gram-negative pathogens more frequently and reliably (at genus and species level) in blood culture broths, this would suggest it has a more valuable role in the identification of gram-negative organisms at present.

The shortcomings of MALDI-TOF MS in the direct identification of blood culture pathogens include the poor identification of polymicrobial bacteremias, α-haemolytic streptococci and anaerobes [11], [22], [23]. Although MALDI-TOF MS can accurately identify one organism in polymicrobial bacteremias (64.5% in the present study), it does not reliably identify the others that are present. This suggests that MALDI-TOF MS is not useful for the direct identification of all organisms from blood culture broths when polymicrobial bacteremia is suspected based on gram stain results, but can still used for the identification of individual isolates following subculture. We report similar findings of poor performance of MALDI-TOF MS in identifying anaerobes and the misidentification of *S. oralis*/*mitis* as *S. pneumoniae* [9], [11], [14]. Although optochin susceptibility and bile solubility can be used to differentiate *S. oralis*/*mitis* from *S. pneumoniae* isolates, this is not possible in the direct identification of organisms from blood culture broths. However, the detection of *S. pneumoniae* polysaccharide cell wall antigen may help confirm direct identification of *S. pneumoniae* from blood culture broths, although false positives can occur with viridans group streptococci, particularly *S. mitis* [17].

There were several limitations in the present study, including the lack of specificity data, as only positive blood culture broths were examined. We were unable to assess if the rapid provision of information about pathogen identification to clinicians affected the management of individual patients, and if patient outcomes and infection control protocols of patients with multi-resistant organisms (such as MRSA) were improved. Nevertheless, we have shown that the MALDI Sepsityper™ Kit and MALDI-TOF MS is a rapid and accurate method for the identification of pathogens from positive blood culture broths. Variations in methodology of blood culture broth processing should be taken into consideration when interpreting results from this and other studies. The identification of gram-positive bacteria should improve with on-going technical development and further refinement of the reference spectra within the MALDI Biotyper database. In future, the identification of the etiologic pathogen in bacteremic patients presenting with shock within the critical six hours will further reduce morbidity and mortality.
